# Maternal phenotype, independent of family economic capital, predicts educational attainment in lowland nepalese children

**DOI:** 10.1002/ajhb.22852

**Published:** 2016-05-02

**Authors:** Akanksha A. Marphatia, Delan Devakumar, Jonathan C.K. Wells, Naomi Saville, Alice Reid, Anthony Costello, Dharma S Manandhar, David Osrin

**Affiliations:** ^1^ Department of GeographyUniversity of CambridgeUnited Kingdom; ^2^ Institute for Global Health, University College LondonLondonUnited Kingdom; ^3^ Childhood Nutrition Research Centre, University College London Institute of Child HealthLondonUnited Kingdom; ^4^ Mother and Infant Research ActivitiesKathmanduNepal

## Abstract

**Objectives:**

Factors acting before children are born or reach school‐going age may explain why some do not complete primary education. Many relevant factors relate to maternal phenotype, but few studies have tested for independent associations of maternal factors relative to those characterizing the family in general.

**Methods:**

Using data from a longitudinal study of 838 children in Dhanusha, Nepal, we used logistic regression models to test whether indices of maternal somatic and educational capital, or family economic capital, were independently associated with children having had ≤2 versus 3+ years of schooling at a mean age of 8.5 years. We also tested whether maternal age, children's early growth, and urban/rural location mediated such associations.

**Results:**

Children had a higher risk of completing less schooling if their mothers were short, thin, anemic, and uneducated. Independently, lower family material assets and land acreage also increased children's odds of less schooling. There was an indication of gender differences, with the risk of poor educational attainment in girls associated with low maternal somatic and educational capital, whereas in boys the relevant factors were low maternal education and family land ownership.

**Conclusions:**

Our analysis demonstrates that, independent of broader indices of family capital such as land or material assets, children's educational attainment is associated with factors embodied in maternal phenotype. Both somatic and educational maternal capital appeared important. A composite index of maternal capital could provide a new measurable proxy, prior to school entry, for identifying children at risk of completing fewer years of schooling. Am. J. Hum. Biol. 28:687–698, 2016. © 2016 Wiley Periodicals, Inc.

International human rights law guarantees the right to education (*Universal Declaration of Human Rights*, 1948). Education is increasingly understood to benefit many outcomes, including health, earning potential and women's decision‐making power (Colclough et al., 2010; Pridmore, 2007; Smith et al., 2003). Recognizing this entitlement, and the potential benefits of education, world leaders made universal primary education and gender equality two of the eight Millennium Development Goals (United Nations General Assembly, 2000). In 2015, world leaders reaffirmed in the Sustainable Development Goals their commitment to achieving these two global priorities by 2030 (United Nations General Assembly, 2015).

It is, therefore, of major concern that, in 2013, the total number of children and adolescents out of school totaled 124 million (UIS and UNICEF, 2015). To address this, diverse policies and interventions have targeted school‐based constraints such as teaching and learning practices (UNESCO, 2015). These efforts have focused on increasing the two complementary benefits of schooling: educational attainment (years of schooling completed) and achievement (performance) (UIS, 2012). Although exam performance is one of the best ways to rank children's achievement, from a policy perspective it is essential to start by ensuring participation in education.

Despite these efforts, many children are still not in school (UIS and UNICEF, 2015). Crucially, children out of school are essentially ‘invisible’ in conventional educational research, because either they never attended, or the system lost track of them after they left. Hence, the broader factors associated with poor attainment are not identified in conventional research, which instead addresses school‐based factors. This means that school‐based interventions may simply be too late for many children.

In the biomedical field, studies in low‐ and middle‐income countries have shown that components of children's phenotype measured *before* they are of school‐going age predict their educational outcomes. In particular, poor infant growth and nutritional status have emerged as key parameters in this context. The large COHORTs study of 8,362 children in Brazil, Guatemala, India, the Philippines, and South Africa found that higher birth weight, faster linear growth, and greater relative weight at 2 years were each associated with an increased likelihood of completing secondary school (Adair et al., 2013; Daniels and Adair, 2004; Maluccio et al., 2009). In Brazil, Guatemala, and the Philippines, stunted children completed an average 0.9 fewer years of education and had a 16% increased risk of failing at least one grade, compared with their nonstunted peers (Martorell et al., 2010). Another study estimated a 7.9% decrease in the likelihood of completing primary education for every 10% increase in the prevalence of stunting (Grantham‐McGregor et al., 2007). Similar findings have been reported from Ghana and Tanzania (Beasley et al., 2000; Fentiman et al., 2001; The Partnership for Child Development, 1999). In a review of African‐American populations, Crooks (1995) found that low birth‐weight, poor growth, and ill‐health were likely to lead to low school achievement, school absenteeism and drop‐out. In Guatemala, nutritional supplementation in the early years had beneficial effects on women's educational achievement (Li et al., 2004). Collectively, this work highlights the benefits of early growth and nutrition for educational outcomes, and the potential role of maternal phenotype.

It is, however, still difficult to predict in advance who will do less well at school. We hypothesize that, independent of broader family circumstances, assessment of maternal phenotype prior to age of school entry could improve the identification of children at risk of completing less education. This approach could also identify potential targets for interventions to increase the years of schooling completed by children.

### Maternal effects

Mothers can potentially influence their offspring by several different pathways. The importance of maternal pregnancy energy transfer is demonstrated by widespread findings linking low birth weight with subsequent underweight, stunting, and wasting in the first 5 years (Paul et al., 2011; WHO, 2006). Indeed, the process of stunting may reflect persistent, cumulative effects of poor nutrition spanning several generations (Dewey and Begum, 2011; Özaltin et al., 2010; Subramanian et al., 2009). In turn, maternal malnutrition is an important predictor of low birth weight (Fall et al., 1998; Veena et al., 2010). Inadequate fetal nutrition may specifically affect the brain: in Scottish school children, gestational age broadly showed an inverse dose‐response relationship with the risk of cognitive impairment, pre‐sumably because preterm birth prevents a delivery of adequate nutrients for brain growth during the last weeks of pregnancy (MacKay et al., 2010). Finally, macronutrients such as specific fatty acids, and micronutrients such as iron or zinc, may be important for fetal brain development (Black, 2003; Grantham‐McGregor and Ani, 2001; Nyaradi et al., 2013).

Whilst the association between maternal phenotype and children's early growth is, therefore, well established, and early life nutrition has been linked with educational success (Adair et al., 2013; Maluccio et al., 2009), little is known about how maternal nutritional status directly predicts children's educational outcomes (Walker et al., 2011).

Beyond nutritional pathways, higher levels of maternal education are associated with better nutritional and educational outcomes in children (Govinda and Bandyopadhyay, 2008; Ruel and Alderman, 2013; Schultz, 2002). Similarly, studies also identify higher maternal socio‐economic position, financial autonomy, and greater decision‐making as key predictors of better developmental outcomes in children (Barros et al., 2010; Chevalier and O'Sullivan, 2007; Shroff et al., 2011; Smith and Haddad, 2000; Victora et al., 1987).

Although this research is important, no study has adopted a more comprehensive approach, simultaneously testing associations of multiple components of maternal phenotype with children's educational attainment, whilst also taking into account broader family circumstances. To address this issue, we developed an approach based on the concept of maternal capital.

### Conceptual model

The maternal capital model was developed from the broader evolutionary “embodied capital” approach of Kaplan and colleagues (Hill and Kaplan, 1999; Kaplan et al., 1995). These authors proposed that individuals invest over the life‐course in a “stock” of capital, including strength, immune function, coordination, skill, and knowledge to maximize reproductive success (Hill and Kaplan, 1999). Males and females may accumulate different types of capital, reflecting the way that they maximize reproductive fitness through different strategies (Wells, 2012). From a societal perspective, this means that those subjected to social and economic exploitation and disempowerment over the life‐course may pay penalties in these traits, ultimately threatening their chances of reproductive and social success.

Building on this approach, Wells (2010) defined maternal capital as aspects of somatic, educational, cultural and material or financial resources enabling differential investment in offspring. The model emphasizes that during fetal life, and to some extent in infancy, offspring in most mammal species are not exposed to the external environment directly, but to maternal phenotype, which embodies maternal capital (Wells, 2010, 2003). On this basis, maternal factors acting within early “critical windows” of physiological sensitivity (Lucas, 1991) are predicted to have greater influence on offspring than those acting in later developmental periods (Wells, 2014, 2003,). This is because many traits are expected to become canalized, that is, less responsive to environmental stresses, when the influence of maternal capital ends (Wells, 2014, 2003).

The benefits to the offspring of exposure to maternal capital during early life clearly depend on the magnitude of the capital. While offspring exposed to high levels may reap life‐long phenotypic benefits, those exposed to low levels may bear life‐long costs. Our conceptual model, therefore, provides a novel approach to understanding how poverty and adversity on the one hand, and public health interventions on the other hand, might impact the next generation via the conduit of maternal capital.

To put maternal capital in context, it is helpful to also measure broader components of family capital—for example, economic capital—such as material assets and land ownership. Other studies have identified lower household socio‐economic position, especially in rural areas, with poor educational attainment (Bhaumik and Chakrabarty, 2013; Reed et al., 1996). These factors are less closely associated with maternal phenotype and are expected to have no opportunity to influence maternal development prior to marriage. Whether economic capital is considered family or maternal capital may also depend on who controls them. Rather than simply adjusting for this variable in analyses, it would be useful to examine it in greater detail. We use this broader approach to explore variability in children's educational attainment in Nepal (Fig. 1).

**Figure 1:**
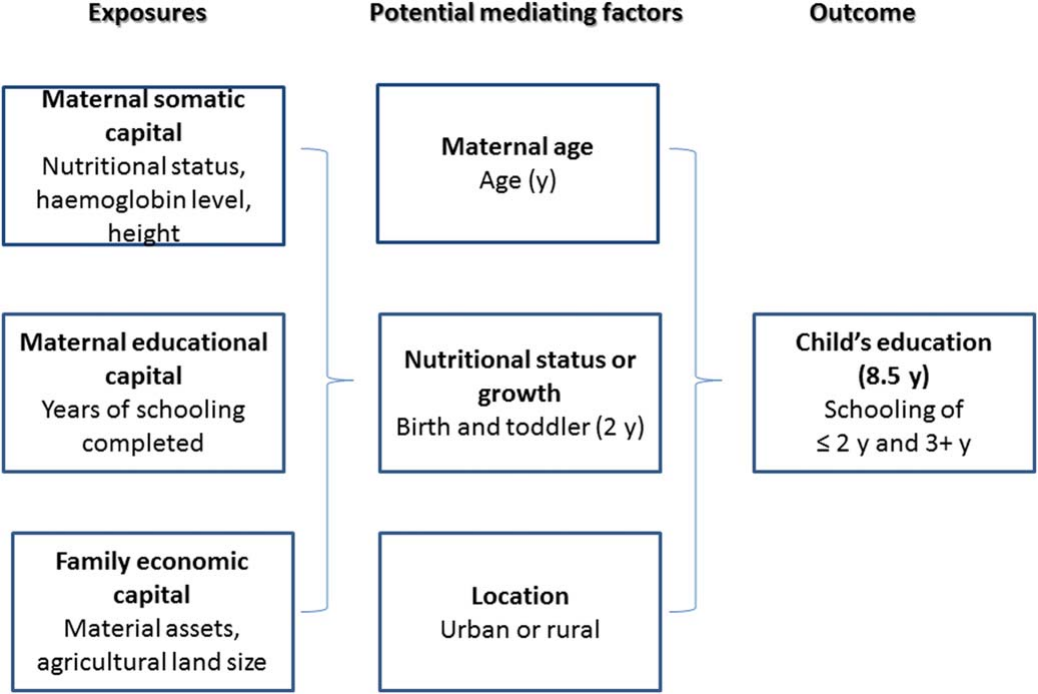
Life‐course maternal capital model.

### The context in Nepal

Access to education has improved in Nepal, reflecting government policy promoting universal basic education, including “early childhood education and development” (ECED) and special initiatives supporting equity and social inclusion (Department of Education, 2013; Ministry of Education Nepal, 2009). However, not all children are enrolled in ECED (age 4 years), or complete their basic education (primary schooling of 5 years, from ages 5 to 9 years, and lower secondary of 3 years, from ages 10 to 12). In 2013, the percentage of new entrants in primary grade 1 with ECED experience was 57% and the net enrolment rate (NER, total number of students of official primary school age enrolled in education, expressed as a percentage of the corresponding population) for primary school was 96% and for lower secondary school 73% (Department of Education, 2013). The percentage expected to complete all five years of primary education was 60% (World Bank and UNESCO Institute of Statistics, 2013).

Although an equal number of girls and boys were *enrolled* in primary education, studies have found that girls’ *attendance* and *completion* were more irregular than boys’ (World Bank and UNESCO Institute of Statistics, 2013). Current efforts to resolve this gender difference by addressing school‐based factors are not proving sufficient to address wider social and gendered norms and geographical constraints (Ayral, 2014; Unterhalter, 2006). It is, therefore, important to identify broader factors, outside the education sector, contributing to these major deficits in children's education.

In keeping with studies elsewhere, high rates of child under‐nutrition in Nepal have also been found to contribute to poor schooling (Moock and Leslie, 1986). The 2011 Nepal Demographic and Health Survey found that 29% of children under 5 years were underweight, 41% were stunted and 11% were wasted (MOHP Nepal et al., 2012). Rates of child malnutrition were more likely to increase with low maternal educational attainment and nutritional status, and poor economic position of families (MOHP Nepal et al., 2012). However, the independent association of each of these maternal capital and family economic components, and children's early growth with their educational outcomes has not yet been investigated.

### Aims

Our goal in this analysis was to investigate the independent associations of maternal and family capital components, assessed before a child's birth, with subsequent years of schooling. We investigated whether low maternal somatic and educational capital components, and low family material assets and land ownership, were independently associated with the likelihood of children attaining insufficient education at a mean age of 8.5 years. We tested whether these associations were mediated by maternal age, child's birth weight, and early growth. Given the high levels of gender inequality in Nepalese society (though not necessarily in basic education), we investigated whether there were differences in educational attainment between girls and boys, and whether the components of capital that increased the likelihood of poor educational attainment differed between the sexes.

We used data from a longitudinal sample of 838 children in Dhanusha District. Their mothers were recruited in 2002–2003 into a randomized control trial of an antenatal multiple micronutrient supplement to investigate the relationship between maternal nutrition and child health (Osrin et al., 2005). Our data pertain to maternal and family capital at birth, child growth assessed at birth and 2 years, and child educational attainment assessed at 8.5 years (Devakumar et al., 2014; Vaidya et al., 2008). Our analysis is observational.

## SUBJECTS AND METHODS

The study was conducted in Dhanusha district in the lowland Central Terai region. In 2013, the Human Development Index for Nepal was 0.54, ranking 145th out of 187 countries. The Gender Inequality Index (a measure of women's status in reproductive health, empowerment, and economic participation) was 0.48, ranking 98th out of 187 countries (UNDP, 2014). Mean life expectancy was 68.4 years and Gross National Income (Purchasing Power Parity, in international dollars for the year 2011) was 2194 (1,857 for females and 2,554 for males).

Details of the trial have been described elsewhere (Osrin et al., 2005). Briefly, 1200 women attending Janakpur Zonal Hospital for antenatal care were recruited. They were randomly allocated to receive either a multivitamin and mineral supplement or a control supplement of iron and folic acid. The supplements were taken daily from between 12 and 20 weeks gestation to delivery and women were assessed every two weeks. Exclusion criteria included multiple pregnancies, fetal abnormalities on obstetric ultrasound, and maternal illness that could compromise the outcome of the pregnancy.

A total of 1,069 mothers and infants completed the trial. Infants were assessed at birth and at 1 month, 2 years, and 8.5 years. Signed informed consent was given in the local language by parents or guardians. The study was registered as an International Standard Randomized Controlled Trial (ISRCTN88625934). The original trial, 2‐year and 8.5‐year follow‐ups were approved by the Nepal Health Research Council (reference 51/2011) and by the University College London (UCL) ethics board (reference 2744/001). The trial was undertaken in collaboration with the Nepal Government Ministry of Health.

Maternal data were collected prospectively and are described in detail by Osrin et al. (2005). Anthropometry of mothers and children was conducted in accordance with UCL Institute of Child Health guidelines, adapted from Lohman et al (1988) and the World Health Organization (WHO) Multi‐Centre Growth Reference Study Group (2006). Body composition in children was estimated with bioelectrical impedance (BC418MA instrumentation, Tanita Corp, Japan), and validated against isotope dilution in this population (Devakumar et al., 2015). Number of years of schooling, socio‐economic status, and land ownership were collected through an oral questionnaire. The questionnaire was developed in Maithili, Nepali, and English, back‐translated to ensure equivalence, piloted in the local population, and adapted prior to use.

Details of children's measurements at the 8.5 year follow‐up are described by Devakumar et al. (2014). Data were collected over 15 months (from September 2011 to December 2012). Children were a mean age of 8.5 years (ranging from 7.2 to 9.9 years). Every effort was made to locate all children from the original trial using geo‐locational data. The 8.5‐year follow‐up included 841 children. The majority of the 228 children not included were lost to follow‐up, but 45 had died and 7 were unable or unwilling to attend for anthropometry.

### Variables

#### Exposures

Our exposures were different components of maternal capital. Maternal somatic capital was defined as height (cm), blood hemoglobin level (g/dl) and body mass index (BMI, weight/height^2^ in kg/m^2^) at time of enrolment in the study (mean gestation of 15 weeks). We tested all continuous variables for linear associations with the outcome. We created tertiles for height and BMI based on previous maternal anthropometric studies (Subramanian et al., 2009). This approach categorized maternal short stature as <146.9 cm, and tall stature as >153.1 cm. Low BMI was <18.5 cm/kg^2^ and high BMI >20.5 cm/kg^2^. When BMI was used as a continuous variable, it was transformed to logarithms for use in statistical tests as it was positively skewed. Hemoglobin levels were dichotomized as anemic (<12 g/dl) or non‐anemic according to the WHO classification (WHO, 2011). Years of schooling were categorized into three levels according to the Nepalese education system: none, primary (1–5 years), and secondary or higher (6+ years) (Ministry of Education Nepal, 2009).

Our total sample size was 838. We omitted three mothers from our analysis. Two were extreme outliers, one for BMI of 8 standard deviation scores (SDS), and one for height of > ‐4 SDS. The other mother had inconsistent values for height at two time points that could not be resolved.

For economic status, we considered levels of material assets and land ownership. We identified these as ‘family’ economic capital since their availability to a mother is accessed through marriage and they may be under paternal/patriarchal control. For material assets, a score was obtained using questionnaire data collected in pregnancy. Households were ranked according to the material assets they possessed using predefined criteria set by the WHO. The questions stratified households into four categories, with more expensive items like a motor vehicle or a refrigerator given the highest ranking; hand tractor, sewing machine or fan the higher‐middle ranking; a clock, radio or bicycle the lower‐middle ranking; and none of these items the lowest rank (WHO SEARO, 1994). From these four categories, we combined the two medium‐ranked groups, which contained very few people, creating three groups for our logistic regression models. Family land ownership was also assessed by questionnaire in terms of area using local units (1 dhur = 0.004 acres or 3.6 square feet). Land ownership was positively skewed and natural log‐transformed for use in statistical tests, but reported as the untransformed value.

#### Outcome

**Figure 2:**
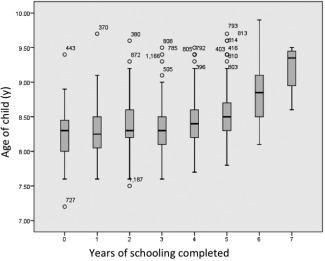
Child's age by years of schooling.

In Nepal, children aged 8 years are expected to have completed 3 years of schooling, and each additional year of age increases that expectation by another year of schooling (Ministry of Education Nepal, 2009). Child age correlated with years of schooling (Fig. 2). In our sample, 88.7% of children were aged 8 years or older. For our outcome variable, we therefore divided our sample into two groups according to whether children had completed ≤2 years or 3+ years of schooling. Based on this approach, our groups comprised 143 with ≤2 years and 695 with 3+ years of education. We considered child age as a potential confounding factor, because of the variability in age (7.2–9.9 years) and its correlation with years of schooling. Children with 3+ years of schooling might either be 9 years old, or have attended pre‐primary education, or have repeated a standard. In each case, we assumed that more years of schooling was a better outcome than fewer years. Even if children had repeated a year, this was considered better than having dropped out of school.

#### Mediators

For our mediating variables, we included maternal age (*y*) as a continuous variable and we dichotomized location as urban or rural. We converted children's growth variables to age‐ and sex‐specific *z*‐scores (SDS) using WHO reference data, using the Lambda, Mu Sigma Method (LMS) option in Microsoft Excel for age‐related reference ranges (Cole and Green, 1992). We computed conditional *z*‐scores for child weight and height at age 2 years, adjusted for size at birth. These conditional *z*‐scores evaluate size at 2 years relative to what was expected for size at birth (Adair et al., 2009).

### Statistical analysis

Our analysis was conducted in SPSS 21. First, we tested whether maternal, family and child capital differed by gender. Then we quantified differences in maternal and child capital components between groups, differentiated in terms of children's educational attainment. We used chi‐square and independent samples t‐tests to test for gender differences and whether groups of children with ≤2 years versus 3+ years of schooling differed in terms of their maternal somatic and educational capital, or family economic capital. We also tested for correlations between components of maternal and family capital and whether they differed by urban or rural residence.

We developed two multivariable logistic regression models. First, we quantified the risk or probability, expressed as Odds Ratios (OR), of attaining less education associated with low maternal or family capital. Our reference group was the highest level of these capital components. We investigated which maternal and family economic capital components independently increased the risk of children attaining fewer years of schooling. We report results with and without land ownership because, although not predictive of children's schooling in the combined model, it did predict boys’ schooling. Our second model investigated whether maternal age, urban or rural location, and children's early growth mediated these associations.

In both logistic regression models we initially controlled for child's age and sex. We also stratified by sex to investigate if different capital components independently increased the risk of low schooling for girls and boys. However, as our sample was smaller when stratified by sex, these results are less robust. The NagelKerke value, a pseudo R^2^ measure (Kirkwood and Sterne, 2003), was multiplied by 100 to show the percentage of variability in children's schooling explained by each model.

## RESULTS

There were some small biases between the 838 children followed and the 362 lost to follow‐up **(**Supporting Information Table 1). Mothers of children lost to follow‐up had higher BMI and had completed more years of schooling. Compared with those followed, children lost to follow‐up had slightly different proportions of ethnicities and religious affiliations, and were more likely to be from urban areas. Preliminary analyses tested whether trial allocation group was associated with any of the exposures or outcomes. As no differences were found between the two trial arms (intervention and control group), or by ethnicity or religious affiliation, no adjustments were made in further modeling. However, rural residency mediated the association between maternal education, assets and land ownership, and was maintained as a mediating factor in our second logistic model.

### The life‐course maternal capital model

There were modest correlations between some components of maternal and family capital (Supporting Information Table 2), indicating that mothers with more schooling were also taller and heavier, and had more assets. A greater proportion of mothers and children living in rural areas attained fewer years of schooling than those in urban areas. There was differential importance of the socio‐economic variables in rural and urban areas. Rural families owned fewer material assets but more land than those in urban areas (Supporting Information Table 3). On average, both girls and boys had low z‐scores for weight, height and BMI relative to WHO reference data **(**Supporting Information Table 4). The absolute sex differences in children's size and body composition, present at birth and at age 2 years, became minimal when expressed in *z*‐score format. There were no significant differences between the sexes in maternal or family capital components (Table 1). Chi‐square tests showed no gender differences in children's years of schooling (Table 2).

**Table 1:** Baseline characteristics of exposures: maternal somatic and educational capital and family economic capital components, stratified by child sex

	All children^a^ (*n* ≥ 829)	Female (*n* ≥ 400)	Male (*n* ≥ 429)
	Mean (SD)	Mean (SD)	Mean (SD)
Age (*y*)	21.6 (3.5)	21.5 (3.4)	21.6 (3.6)
BMI (kg/m^2^)^b^	20.1 (1.1)	20.1 (1.1)	20.1 (1.1)
Height (cm)	151 (5)	151 (5)	151 (5)
Family land ownership (dhur)^b^	4.5 (9.0)	4.5 (9.0)	4.9 (9.0)
	**Frequency (%)** c	**Frequency (%)**	**Frequency (%)**
Anemia			
No (>12 g/dl)	285 (34.4)	144 (36.0)	141 (32.9)
Yes (<12 g/dl)	544 (65.6)	256 (64.0)	288 (67.1)
Years of schooling			
None (0 *y*)	415 (49.5)	196 (48.4)	219 (50.6)
Primary (1–5 *y*)	69 (8.2)	30 (7.4)	39 (9.0)
Secondary or higher (≥6 *y*)	354 (42.2)	179 (44.2)	175 (40.4)
Family material assets (score)			
Low	124 (14.8)	62 (15.3)	62 (14.3)
Medium	285 (34.0)	140 (34.6)	145 (33.5)
High	429 (51.2)	203 (50.1)	226 (52.2)

Abbreviations: SD, standard deviation. Difference between female relative to male.

a Independent samples T‐test showed no difference by sex.

b BMI and land ownership were natural log‐transformed for use in statistical tests but reported as the untransformed value in the table.

c Chi‐squared test showed no differences by sex.

**Table 2:** Baseline characteristics of outcome: Children's educational attainment at 8.5 years, stratified by sex

	All children^a^ (*n* = 838)	Female (*n* = 405)	Male (*n* = 433)
	Frequency (%)	Frequency (%)	Frequency (%)
No. of years of schooling (*y*)			
** **0	14 (1.7)	6 (1.5)	8 (1.8)
** **1	40 (4.8)	20 (4.9)	20 (4.6)
** **2	89 (10.6)	41 (10.1)	48 (11.1)
** **3	201 (24.0)	106 (26.2)	95 (21.9)
** **4	241 (28.8)	118 (29.1)	123 (28.4)
** **5	187 (22.3)	76 (18.8)	111 (25.6)
** **6	62 (7.4)	35 (8.6)	27 (6.2)
** **7	4 (0.5)	3 (0.7)	1 (0.2)
Schooling years, categorized			
** **≤2 *y*	143 (17.1)	67 (16.5)	76 (17.6)
** **3+ *y*	695 (82.9)	338 (83.5)	357 (82.4)

a Chi‐square test showed no differences by sex.

By independent samples *t*‐tests and chi‐square tests, when compared with those with 3+ years of schooling, children with ≤2 years of schooling had mothers who were older on average **(**Table 3 **)**. Children with less schooling had a greater proportion of mothers who were shorter in stature, had lower BMI, were anemic, and had lower levels of schooling. In the whole sample, fewer mothers had had primary education than either none or secondary/higher education. A greater proportion of the families of children with less schooling were categorized in the lower material asset group and owned less land.

**Table 3:** Maternal somatic and educational capital, and family economic capital, stratified by child's schooling (≤2 years vs 3+ years) at 8.5 years

	≤2 *y* schooling (*n* > 138)	3+ *y* schooling (*n* > 655)	Difference^a^
	Mean (SD)	Mean (SD)	Δ (s.e.)
Age (*y*)	22.6 (4.1)	21.4 (3.4)	**1.2 (0.4)** ***
Family land ownership (dhur)^b^	2.4 (11.0)	4.9 (8.2)	**0.5 (1.2)** **
	**Frequency (%)**	**Frequency (%)**	**Significance** c
**Maternal somatic capital**			
Height			**≤0.001**
** **Short (<146.9 cm)	49 (34.3)	143 (20.6)	
** **Average (147.0**–**153.0 cm)	57 (39.9)	306 (44.1)	
** **Tall (>153.1 cm)	37 (25.9)	245 (35.3)	
BMI			**≤0.01**
** **Low (<18.50 kg/m^2^)	57 (39.9)	196 (28.2)	
** **Average (18.50**–**20.49 kg/m^2^)	55 (38.5)	265 (38.2)	
** **High (>20.50 kg/m^2^)	31 (21.7)	233 (33.6)	
Hemoglobin			**≤0.05**
** **Anemic (<12 g/dl)	104 (73.2)	440 (64.0)	
** **Not anemic (≥12 g/dl)	38 (26.8)	247 (36.0)	
**Maternal educational capital**			
Years of schooling			**≤0.001**
** **No education (0 *y*)	112 (78.3)	303 (43.6)	
** **Primary (1–5 *y*)	9 (6.3)	60 (8.6)	
** **Secondary^+^ (≥6 *y*)	22 (15.4)	332 (47.8)	
**Family economic capital**			
Material assets (score)			**≤0.001**
** **Low	38 (26.6)	86 (12.4)	
** **Medium	62 (43.4)	223 (32.1)	
** **High	43 (30.1)	386 (55.5)	

Abbreviations: SD, standard deviation; Δ, difference; s.e., standard error. Difference ≤2 *y* relative to 3+ *y* of schooling.

a Differences calculated by independent samples T‐Test.

b Land ownership were natural log‐transformed for use in statistical tests but reported as the untransformed value in the table.

c Differences calculated by chi‐square test.

^*^ *p* ≤ 0.05, ^**^ *p* ≤ 0.01, ^***^ *p* ≤ 0.001.

In logistic regression models, lower levels of maternal somatic and educational capital, and low family material assets independently increased children's risk of low schooling. Table 4 **(**Model 1, without land ownership**)** shows that children had a higher risk of attaining less schooling if mothers were shorter, of low BMI, anemic, and had no education. Children also had a higher risk of attaining less schooling if their families had lower or average levels of material assets. Gender did not predict schooling, but the risk of low schooling decreased as children got older. This model explained 18.2% of the variance in years of schooling attained by children. Sensitivity analysis showed that excluding children with more than 6 years of education did not change the findings.

**Table 4:** Multivariable logistic regression testing independent associations of maternal somatic and educational capital and family economic capital components with children's odds of ≤ 2 years schooling at 8.5 years, stratified by sex

	Model 1	Model 2	Model 3	
	Without land ownership (*n* = 828)^a^	With land ownership (*n* = 784)^b^	Female (*n* = 405)^c^	Male (*n* = 417)^d^
	(NK = 0.182)	(NK = 0.177)	(NK = 0.166)	(NK = 0.200)
	Exp B (CI)	Exp B (CI)	Exp B (CI)	Exp B (CI)
Child's age (*y*)	**0.4 (0.2, 0.8)** **	**0.5 (0.3, 0.9)** *	0.7 (0.3, 1.5)	**0.3 (0.1, 0.7)** **
Sex (Male = Ref.)	1.0	1.0		
** **Female	1.0 (0.6, 1.4)	1.0 (0.7, 1.5)		
**Maternal capital**				
Height (cm) (Tall = Ref.)	1.0	1.0	1.0	
** **Short	**2.0 (1.2, 3.3)** **	**1.8 (1.1, 3.1)** *	**3.5 (1.6, 7.7)** **	
** **Average	1.1 (0.7, 1.7)	1.0 (0.6, 1.7)	**2.4 (1.1, 5.0)** *	
BMI (kg/m^2^) (High = Ref.)	1.0	1.0	1.0	
** **Low	**1.9 (1.1, 3.1)** *	**1.8 (1.1, 3.0)** *	**2.3 (1.1, 4.8)** *	
** **Average	1.5 (0.9, 2.5)	1.5 (0.9, 2.4)	1.7 (0.8, 3.4)	
Anemia (g/dl) (No=Ref.)	1.0	1.0		
** **Anemic	**1.5 (1.0, 2.4)** *	1.5 (1.0, 2.4)		
Education (*y*) (Secondary+ = Ref.)	1.0	1.0	1.0	1.0
** **None	**3.5 (2.1, 6.0)** ***	**3.4 (2.0, 5.8)** ***	**4.2 (2.1, 8.4)** ***	**5.0 (2.4, 10.4)** ***
** **Primary	1.6 (0.7, 3.7)	1.6 (0.7, 3.7)	2.0 (0.6, 6.8)	1.8 (0.6, 5.9)
**Family economic capital**				
Material assets (score) (High = Ref.)	1.0	1.0		
** **Low	**1.9 (1.1, 3.3)** *	**2.0 (1.2, 3.5)** *		
** **Middle	**1.6 (1.0, 2.6)** *	1.6 (1.0, 2.5)		
Land ownership (dhur)^e^		1.0 (0.9, 1.1)		**0.8 (0.7, 0.9)** **
Constant	27.8	14.9	0.6	2249

Abbreviations: NK, Nagelkerke (pseudo *R^2^*), CI, 95% Confidence Interval.

a Sample size (*n* = 142 ≤ 2 *y*, *n* = 686 3+ *y*,).

b Sample size (*n* = 137 ≤ 2 *y*, *n* = 647 3+ *y*).

c Sample size (*n* = 67 ≤ 2 *y*, *n* = 338 3+ *y*).

d Sample size (*n* = 73 ≤ 2 *y*, *n* = 344 3+ *y*).

e Land ownership were natural log‐transformed for use in logistic regression.

^*^ *p* ≤ 0.05, ^**^ *p* ≤ 0.01, ^***^ *p* ≤ 0.001.

The logistic model in Table 4 **(**Model 2**)** adjusted for land ownership. The coefficients for maternal and family capital components changed negligibly. As in Model 1, low levels of maternal somatic and educational capital and low family material assets independently increased children's risk of low schooling. Maternal anemia was maintained in the model (*p* = 0.061). Neither gender nor land ownership were significant predictors of schooling. This model explained 17.7% of the variance in the years of schooling attained by children.

Different maternal capital components were independently predictive of girls’ and boys’ education (Table 4, Model 3**)**. For girls, the risk of lower schooling was greater when their mothers had shorter or average stature, lower BMI, and no education. This model explained 16.6% of the variance in education attained by girls. For boys, the risk of lower schooling was greater when mothers had no education. The risk of less education decreased as family land ownership increased and as boys got older. This model explained 20.0% of the variance in education attained by boys.

### Mediating variables: maternal age, location, and child growth and body composition at birth and 2 years

Our potential mediating variables were maternal age, location, and growth variables. Our analysis showed that maternal age was not a significant mediating factor, but was instead independently associated with children's education. Rural children were more likely to be in the less educated group. When compared with children with 3+ years of schooling, those with less schooling had a smaller head circumference at birth and were smaller at the age of 2 years (Table 5 **)**. Growth differences in early life tracked on, and less educated children remained shorter and thinner at the age of 8.5 years.

**Table 5:** Child anthropometry stratified by schooling of ≤ 2 vs. 3+ years at 8.5 years

	≤2 *y* schooling (*n* ≥ 115)	3+ *y* schooling (*n* ≥ 511)	Difference^a^
	Mean (SD)	Mean (SD)	Δ (s.e.)
Size at birth			
Weight (kg)	2.7 (0.4)	2.8 (0.4)	−0.1 (0.04)
Length (cm)	48.5 (2.5)	48.9 (2.8)	−0.4 (0.2)
Head circumference (cm)	33.3 (1.6)	33.8 (2.3)	**−0.5** (**0.2**)**
Size at 2 y			
Weight z‐score	−2.9 (0.9)	−2.1 (1.1)	**−0.8** (**0.1**)***
Conditional weight *z*‐score	−0.5 (0.9)	0.1 (0.1)	**−0.6 (0.1)** ***
Height *z*‐score	−2.1 (1.0)	−1.6 (1.0)	**−0.5** (**0.1**)***
Conditional height *z*‐score	−0.6 (0.9)	0.1 (1.0)	**−0.7 (0.1)** ***
BMI *z*‐score	−0.3 (1.2)	−0.3 (1.1)	0.01 (0.1)
Head circumference (cm)	46.1 (1.5)	46.6 (1.4)	**−0.4** (**0.1**)**
Body composition at 8.5 *y*			
Weight *z*‐score	−2.6 (0.9)	−1.9 (1.0)	**−0.6** (**0.1**)***
Height *z*‐score	−2.1 (1.0)	−1.4 (0.9)	**−0.7** (**0.1**)***
BMI *z*‐score	−1.8 (0.9)	−1.6 (1.0)	**−0.2** (**0.1**)*
Head circumference (cm)	48.9 (1.6)	49.4 (1.4)	**−0.5** (**0.1**)***
Lean mass (kg)	16.0 (2.0)	17.6 (2.4)	**−1.6** (**0.2**)***
Fat mass (kg)	2.6 (1.1)	3.1 (1.7)	**−0.5** (**0.2**)**

Abbreviation: SD, standard deviation; Δ, difference; s.e., standard error.

Difference ≤ 2 *y* relative to 3+ *y* of schooling.

a Differences calculated by Independent samples T‐Test.

^*^ *p* ≤ 0.05, **p ≤ 0.01, ***p ≤ 0.001.

Size at birth or 2 years were neither independent predictors nor mediators. Although conditional weight *z*‐score at 2 years was a significant predictor, its contribution was lost in a model that included conditional height *z*‐score at 2 years. This indicates that early postnatal linear growth was the more important factor **(**Table 6, Model 1) and mediated the associations of maternal height and anemia, both of which lost significance in the model. Increasing maternal age was associated with increased risk of less schooling for children. Location mediated the association of family material assets. In this model, land ownership now predicted schooling, with greater acreage decreasing the risk of lower schooling. This model explained 24.4% of the variance in education attained. Sensitivity analysis showed that excluding children with more than 6 years of education did not change the findings.

**Table 6:** Multivariable logistic regression testing if location and child conditional height z‐score at 2 years mediate independent associations of maternal somatic and educational capital and family economic capital components with odds of ≤2 years schooling at 8.5 years, stratified by sex

	Model 1	Model 2	
	All children (*n* = 754)^a^	Female (*n* = 384)^b^	Male (*n* = 397)^c^
	(NK = 0.244)	(NK = 0.321)	(NK = 0.283)
	Exp B (CI)	Exp B (CI)	Exp B (CI)
Child's age (*y*)	0.9 (0.5, 1.8)	1.2 (0.4, 3.1)	0.6 (0.2, 1.5)
Sex (Male = Ref.)	1.0		
Female	1.3 (0.8, 2.0)		
**Maternal capital components**			
Maternal height (cm) (Tall = Ref.)		1.0	1.0
Short		**2.7 (1.1, 6.8)** *	0.9 (0.4, 2.0)
Average		1.9 (0.8, 4.4)	**0.5 (0.2, 0.9)** *
Maternal BMI (kg/m^2^) (High = Ref.)	1.0	1.0	
Low	**1.8 (1.0, 3.0)** *	**2.5 (1.1, 5.8)** *	
Average	1.5 (0.9, 2.5)	2.2 (1.0, 4.9)	
Education (*y*) (Secondary+ = Ref.)	1.0	1.0	1.0
None	**3.0 (1.7, 5.1)** ***	**3.2 (1.5, 7.0)** **	**2.8 (1.2, 6.6)** *
Primary	1.5 (0.6, 3.6)	1.5 (0.4, 5.6)	1.5 (0.4, 5.3)
**Family economic capital**			
Material assets (score) (High = Ref.)			1.0
Low			2.1 (0.9, 4.7)
Middle			2.0 (1.0, 4.0)
Land ownership *(dhur)^d^*	**0.9 (0.8, 0.9)** *		**0.8 (0.7, 0.9)** *
**Potential mediating factors**			
Maternal age (*y*)	**1.1 (1.0, 1.1)** *	1.0 (0.9, 1.1)	**1.1 (1.0, 1.2)** *
Location (Urban = Ref.)	1.0	1.0	
Rural	**1.6 (1.0, 2.5)** *	**2.7 (1.3, 5.6)** **	
Child's conditional height *z*‐score at 2 *y*	**0.5 (0.3, 0.6)** ***	**0.3 (0.2, 0.5)** ***	**0.6 (0.4, 0.9)** *
Constant	0.02	0.002	1.3

Abbreviations: N.K., Nagelkerke (pseudo *R* ^2^); C.I., 95% Confidence Interval.

a (*n* = 134 ≤ 2 *y*, *n* = 620 3+ *y*).

b (*n* = 64 ≤ 2 *y*, *n* = 320, 3+ *y*).

c (*n* = 72 ≤2 *y*, *n* = 325, 3+ *y*).

Land ownership were natural log‐transformed for use in logistic regression.

*p ≤ 0.05, **p ≤ 0.01, ***p ≤ 0.001.

For girls, conditional height at 2 years mediated the association of maternal anemia and average maternal height. Faster linear growth in early childhood decreased the odds of less schooling. Maternal short stature, low BMI, no education and rural residence independently predicted less schooling (Table 6, Model 2**)**. This model explained 32.1% of the variance in education attained by girls. For boys, conditional height at 2 years mediated the association between child age and education. Faster linear growth in early childhood decreased the odds of less schooling. In this Model, maternal average stature and higher land ownership independently decreased boys’ risk of low schooling. The lack of maternal educational capital and higher maternal age independently increased the odds of less schooling. This model explained 28.3% of the variance in education attained by boys.

## DISCUSSION

We investigated whether early life factors, usually not considered in conventional educational research, could predict the risk of low education in children of school‐going age. Previous studies have investigated associations between single components of maternal capital or children's early growth with educational outcomes. Our study goes further by addressing several components of maternal somatic and educational capital, independent of family economic capital. Furthermore, we ran sex‐specific models to provide more information about relevant components of capital for girls and boys.

**Figure 3:**
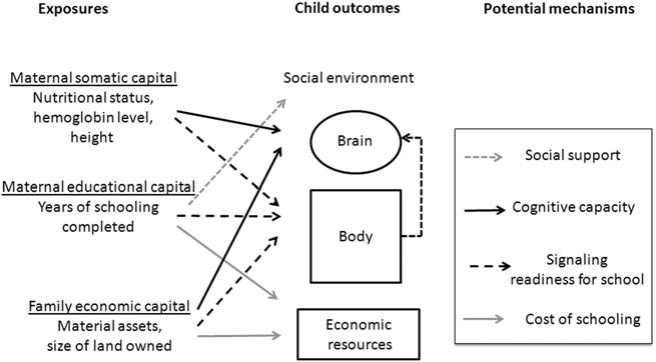
Potential trans‐generational mechanisms.

We found that maternal somatic and educational capital and family economic capital were independently associated with children's schooling. Potential trans‐generational mechanisms are illustrated in Figure 3. Maternal somatic capital may be associated with children's physical growth and cognitive development. Maternal educational attainment may be related to children's learning environment, and together with family economic capital, may also reflect the support provided for education.

These trans‐generational associations are interesting because the different components act as proxies for different life‐course periods of maternal capital accumulation. Maternal height, for example, reflects the health stock accumulated through genes, and social and environmental exposures in early childhood (Özaltin et al., 2010). BMI and anemia, in contrast, reflect current nutritional status of mothers. Maternal somatic capital components may also be proxies for current household economic position and maternal social status within the family. Material assets, which are easily transferable, and ownership of land, a heritable resource, represent contrasting proxies of household wealth. Mothers may not always have access or control over these resources.

Our analysis showed that maternal age was an independent factor predicting children's education, with greater age increasing the risk of the child completing less schooling. This suggests that rather than acting as a proxy for factors such as maternal education and age at marriage (Raj et al., 2014), other characteristics of older mothers seem to constrain the education of their children. For example, there may be a cohort effect, with older mothers inherently placing less value on education for their children. This issue merits further investigation.

Education is usually acquired during childhood and adolescence, but may also reflect the mother's own parents’ attitudes. Studies find that mothers who see the benefits of their own schooling are more likely to promote early school entry, regular attendance, and completion for their children (Caldwell et al., 1983; Govinda and Bandyopadhyay, 2008; Schultz, 2002). This assumes, however, that mothers have control or decision‐making authority over their children's education.

Potential underlying pathways for some of our factors, such as maternal anemia, require further investigation (Walker et al., 2011). There is growing evidence that anemia in early childhood is associated with poor cognitive development. Iron supplementation trials in early childhood have shown improved intellectual functioning, cognition, and educational achievement in Nepal (Christian et al., 2010), Indonesia (Idjradinata and Pollitt, 1993; Soemantri et al., 1985) and India (Seshadri and Gopaldas, 1989).

Our multicomponent approach helps us understand how the cumulative acquisition of maternal and family capital through the life‐course is associated with educational attainment in the offspring. In turn, this helps understand which factors are most amenable to short‐term interventions, and which factors require longer‐term perspectives.

Children with less schooling were smaller at birth and at 2 years. Our finding that conditional height at 2 years mediated the associations of maternal height and anemia supports previous studies on the importance of growth in early postnatal life (Adair et al., 2013; Martorell et al., 2010; The Lancet Maternal and Child Nutrition Study Group, 2013). Research from Jamaica (Walker et al., 1996), Kenya (Mukudi, 2003), Guatemala (Brown and Pollitt, 1996), Ghana (Fentiman et al., 2001), and Tanzania (Beasley et al., 2000) offers several explanations for the association of this nutritional deficit with education. Children who are perceived as being “small” for their age may also be perceived as not being “ready” for school. Research suggests that parents often send them to school at an older age (Brown and Pollitt, 1996). Whether such children are also genuinely slower in their development requires further investigation, but growth retardation, indicating a lack of nutrients at the cellular level in early life, has been associated with poorer brain and neurological development (Martorell et al., 2010).

Poor early growth of children may thus be a composite marker for doing less well in school, although, independent of this, children's education was still predicted by other components of maternal capital. Here, Crooks (1997) reminds us that the association between child growth and education is complex; to understand it requires focusing on the conditions in the environment that produce poor growth. Our analysis has focused on the associations of low maternal somatic and educational capital, and household economic assets with low educational attainment.

In our cohort, educational and nutritional disparities between the sexes were not apparent in early life or at 8.5 years. This is a particularly interesting finding because women's overall status in society in Nepal is low, reflected by its ranking of 98 out of 187 countries on the Gender Inequality Index (GII) (UNDP, 2014). Even if girls’ enrolment in primary school is high, education statistics may not index irregular participation, nor address sufficiently the barriers to entering secondary school (Unterhalter, 2006).

It is possible that gender differences in education may develop at a later age in our cohort. They may also be narrowing in the current generation, perhaps on account of the availability of more and closer educational facilities. Although we did not have data on paternal education or nutritional status, the fact that different components of maternal capital independently predicted more education in children in a patriarchal society suggests the importance of investing specifically in women as one strategy for preventing low education for children.

Interestingly, there was a difference between girls and boys in the capital components predicting low education. Low maternal stature and BMI predicted less education for girls, but not boys. This may indicate that families may perceive girls, particularly those of smaller stature in the early years, less ready for school. The lack of association of maternal somatic capital with boys’ schooling may also be due to male preference, such that families in situations of extreme stress may prioritize sending boys to schools over girls, regardless of maternal capital. Greater land ownership reduced the risk of less education for boys, but not girls. The importance of this family economic capital for boys’ education might be explained by its patrilineal heritability. Land may be considered a proxy for household wealth as shown in another longitudinal analysis of associations of child under‐nutrition with schooling years in the Terai region of Nepal (Jamison and Lockheed, 1987). Mothers living in rural areas had lower levels of education, and rural girls also had a higher risk of less schooling, suggesting that either appropriate school environment or transport may not be readily accessible. This may also indicate lower household investment in facilitating girls’ access to school in rural areas because boys in similar areas did not have disadvantaged access to education. It is also possible that families in rural areas with greater land ownership prefer to send boys rather than girls to school.

There are data‐related limitations to our analysis. We lack data on school attendance, making it difficult to know how many children never attended, or attended irregularly, or dropped out. The education outcome variable, years of schooling, does not necessarily indicate the standard of attainment in school or exam performance. Nevertheless, given the age range of our sample, it was reasonable for us to assume that children should have completed 3+ years of school, and that attaining fewer years indicated lower attainment. We also could not address school‐related factors such as teacher quality or infrastructure.

Our analysis was based on a randomized trial, which aimed to change some components of maternal capital (specifically micronutrient status) during pregnancy. However, whether the child was in the intervention or control arm of the trial did not predict the duration of schooling. Furthermore, according to a chi‐square test, there was no difference in the prevalence of maternal anemia in the two trial arms, indicating that it did not affect the components of capital that were significant in our analysis. Whilst the trial did show an association with weight at 2 years, it did not show an association with height, or change in height after birth (Osrin et al., 2005; Vaidya et al., 2008). Therefore we have no evidence that the trial design confounded our findings.

Finally, potentially important components of the maternal capital exposure may not have been measured, and we cannot discern exactly how or when associations between maternal capital and offspring phenotype emerge. We also lack markers of wider household educational capital such as paternal education and home learning environments (Brown and Pollitt, 1996; Goodman and Greg, 2010). Nevertheless, our data support the hypothesis that maternal capital is an important conduit through which environmental factors shape the educational potential of children during the period of the life‐course prior to school entry.

This period of development is generally missed in conventional education research, which tends to investigate traits in school‐going children. Critically, our analyses demonstrate independent associations between maternal somatic and educational capital components and children's schooling, even after adjusting for birth weight and early growth in our statistical models. The mechanism through which maternal capital benefits child education appears not to be restricted to early growth.

Others concur that the most effective and cost‐efficient period in which to address educational inequalities may be in early life before major nutritional deficits accumulate (Walker et al., 2011). Our findings indicate the importance of maternal capital in such a Developmental Origins model of educational attainment. The importance of breaking this trans‐generational cycle of risk is important for all children, but especially for girls, who may transmit this disadvantage to their own offspring (Schell, 1997). In practical terms, we suggest that identifying mothers with lower somatic, educational, and (household) economic capital could help identify children at risk of less education. If our findings are confirmed in other studies, our theoretical model also suggests potential avenues for intervention.

We suggest that greater coordination between educational and public health nutrition research could lead to future interventions being more successful on both counts. We suggest that interventions specifically benefitting maternal capital are potentially one means of improving children's development and educational attainment in Nepal. An example of a successful approach to increasing children's education, particularly that of girls, is the improvement of maternal literacy as part of wider efforts to support women's groups (ActionAid, 1996; Marphatia and Moussié, 2013). There are clearly major benefits to intervention strategies that are capable of impacting both nutritional status and educational performance of children, and which may also benefit the mothers themselves.

## AUTHOR CONTRIBUTIONS

The original trial was designed and conducted by DO, AC, and DM. The follow‐up at 8 years was designed by DO, DD, JW, NS, and DM. DD developed the measurement protocol and led the fieldwork team. This analysis was designed by AM, DD, JW, and DO. AM conducted the statistical analyses with assistance from AR and JW. AM wrote the first draft of the paper. All authors provided critical comments and helped revise the paper.

## DECLARATION OF INTEREST

Jonathan Wells has previously received funding and two bioelectrical impedance analysis instruments from Tanita UK. This donor had no influence on the design, funding, conduct or interpretation of the present study. The other authors declare no competing interests.

## Supporting information

Supporting Information
