# Supplementary material for: Maternal phenotype, independent of family economic capital, predicts educational attainment in lowland nepalese children
Source: Am J Hum Biol. 2016 May 2;28(5):687–98. doi: 10.1002/ajhb.22852 (PMC5026068; doi:10.1002/ajhb.22852)
Supplement: Supplementary file 1 — Supporting Information [file AJHB-28-687-s001.docx]

| \| Supplementary Table 1. Maternal somatic and educational capital and family economic capital components of 838 children followed-up at 8.5 years and362 lost to follow-up \| \| \| \| \| \| \| --- \| --- \| --- \| --- \| --- \| --- \| \|  \| Lost to follow-up  (n≥236) \|  \| Followed-up  (n≥816) \|  \| Difference^1^ \| \|  \| Mean (SD) \|  \| Mean (SD) \|  \| Δ (s.e.) \| \| Maternal characteristics \| 21.4 (3.6)  20.1 (1.1)  151 (5.6) \|  \| 21.6 (3.5)  20.1 (1.1)  151 (5.3) \|  \| -0.2 (0.2)  **1.0 (1.0)****  -0.003 (0.3) \| \| Age (y) \|  \|  \| \| BMI (kg/m^2^)^2^ \|  \|  \| \| Height (cm) \|  \|  \| \| Family Land ownership (dhur)^2^ \| 4.9 (9.0) \|  \| 4.5 (9.0) \|  \| 1.1 (1.1) \| \| Birth weight of child (kg) \| 2.7 (0.5) \|  \| 2.8 (0.4) \|  \| -0.03 (0.03) \| \|  \|  \|  \|  \|  \|  \| \|  \| **Frequency (%)** \|  \| **Frequency (%)** \|  \| **Significance**^3^ \| \| Maternal capital \|  \|  \|  \|  \|  \| \| Anaemia  No (<12 g/dl)  Yes (>12 g/dl)  Years of schooling (y) \| 116 (33.8)  227 (66.2) \|  \| 285 (34.4)  544 (65.6) \|  \| ns \| \| None (0y)  Primary (1-5 y)  Secondary or higher (6-13 y) \| 129 (35.6)  54 (14.9)  179 (49.4) \|  \| 415 (49.5)  69 (8.2)  354 (42.2) \|  \| **≤0.001** \| \| Family economic capital \|  \|  \|  \|  \|  \| \| Family material assets (score)  Low  Medium  High \| 61 (16.9)  116 (32.1)  184 (51.0) \|  \| 124 (14.8)  285 (34.0)  429 (51.2) \|  \| ns \| \| Family characteristics \|  \|  \|  \|  \|  \| \| Ethnicity  Terai Brahmin  Terai Chhetri  Terai Vaishya  Terai Sudra  Hill Brahmin  Hill Chhetri  Muslim  Newar  Tibeto-Burman group  Other  Religion  Hindu  Muslim  Other  Location  Urban  Rural  District  Dhanusha  Mahottari  Sarlahi  Siraha \| 55 (15.2)  20 (5.5)  217 (59.9)  5 (1.4)  18 (5.0)  7 (1.9)  25 (6.9)  4 (1.1)  10 (2.8)  1 (0.3)  332 (91.7)  25 (6.9)  5 (1.4)  233 (64.4)  129 (35.6)  307 (84.8)  53 (14.6)  1 (0.3)  1 (0.3) \|  \| 105 (12.5)  22 (2.6)  569 (67.9)  24 (2.9)  24 (2.9)  23 (2.7)  52 (6.2)  9 (1.1)  9 (1.1)  1 (0.1)  786 (93.8)  51 (6.1)  1 (0.1)  394 (47.0)  444 (53.0)  681 (81.3)  153 (18.3)  2 (0.2)  2 (0.2) \|  \| **≤0.05**  **≤0.05**  **≤0.001**  ns \|   Abbreviations: SD, standard deviation; Δ, difference lost to follow-up relative to followed-up group; s.e., standard error. ^1^Independent samples t-test. ^2^BMI and land ownership were log transformed for these tests. Unlogged values are reported in the Table. ^3^Chi-square test. **p≤0.01. ns, not significant. |
| --- | --- | --- | --- | --- | --- | --- | --- | --- | --- | --- | --- | --- | --- | --- | --- | --- | --- | --- | --- | --- | --- | --- | --- | --- | --- | --- | --- | --- | --- | --- | --- | --- | --- | --- | --- | --- | --- | --- | --- | --- | --- | --- | --- | --- | --- | --- | --- | --- | --- | --- | --- | --- | --- | --- | --- | --- | --- | --- | --- | --- | --- | --- | --- | --- | --- | --- | --- | --- | --- | --- | --- | --- | --- | --- | --- | --- | --- | --- | --- | --- | --- | --- | --- | --- | --- | --- | --- | --- | --- | --- | --- | --- | --- | --- | --- | --- | --- | --- | --- |

| Supplementary Table 2. Correlations amongst components of maternal somatic and education capital and family economic capital components^1^ | | | | | | |
| --- | --- | --- | --- | --- | --- | --- |
|  | Height  (cm) | BMI^2^  (kg/m^2^) | Haemoglobin  (g/dl) | Education  (y) | Material assets (score) | Land ownership (dhur)^2^ |
| Age (y) | 0.01 | -0.02 | -0.03 | **-0.13*** | -0.05 | -0.03 |
| Height (cm) |  | -0.07 | 0.01 | **0.21*** | **0.10*** | **0.15**** |
| BMI (kg/m^2^)^2^ |  |  | **0.10*** | **0.10*** | **0.10*** | 0.04 |
| Haemoglobin (g/dl) |  |  |  | 0.02 | 0.02 | 0.01 |
| Education (y) |  |  |  |  | **0.45*** | **0.25**** |
| Material assets (score) |  |  |  |  |  | **0.21**** |

^1^ Pearson correlations. ^2^BMI and land ownership were log transformed for these tests. *p≤0.05, **p≤0.01

| Supplementary Table 3. Maternal somatic and educational capital, family economic capital, and child years of schooling, stratified by urban and rural location | | | | | |
| --- | --- | --- | --- | --- | --- |
|  | Urban (n=344) |  | Rural (n=494) |  | Difference^1^ |
|  | Mean (SD) |  | Mean (SD) |  | Δ (s.e.) |
| Maternal age (y)  Family land ownership (dhur)^2^ | 21.7 (3.6)  3.3 (9.9) |  | 21.5 (3.5)  5.9 (7.8) |  | 0.3(0.2)  **-0.5 (1.2)^***^** |
|  |  |  |  |  |  |
|  | **Frequency (%)**  99 (25.2)  168 (42.7)  123 (32.1)  126 (32.1)  148 (37.7)  119 (30.3)  121 (31.2)  267 (68.8)  138 (40.1)  22 (6.4)  184 (53.5) |  | **Frequency (%)** |  | **Significance^3^**  ns |
| **Maternal somatic capital** |  |  | 93 (20.9)  195 (43.9)  156 (35.1)  127 (28.6)  172 (38.7)  145 (32.7)  164 (37.2)  277 (62.8)  277 (56.1)  47 (9.5)  170 (34.4) |  |  |
| Height  Short (<146.9 cm)  Average (147.0-153.0 cm)  Tall (>153.1 cm) |  |  |  |  |  |
|  |  |  |  |  |  |
| BMI  Low (<18.49 kg/m^2^)  Average (18.50 – 20.49 kg/m^2^)  High (>20.50 kg/m^2^) |  |  |  |  | ns |
|  |  |  |  |  |  |
| Anaemia  Not anaemic (>12 g/dl)  Anaemic (<12 g/dl) |  |  |  |  | =0.069 |
|  |  |  |  |  |  |
| **Maternal educational capital** |  |  |  |  | **≤0.001** |
| Years of schooling  No school (0 y) |  |  |  |  |  |
| Primary (1-5 y) |  |  |  |  |  |
| Secondary or higher (<6 y) |  |  |  |  |  |
|  |  |  |  |  |  |
| **Family economic capital**  Family material assets (score)  Low  Medium  High | 34 (9.9)  70 (20.3)  240 (69.8) |  | 90 (18.2)  215 (43.5)  189 (38.3) |  | **≤0.001** |
|  |  |  |  |  |  |
| **Children’s years of schooling** |  |  |  |  | **≤0.01** |
| ≤ 2 y | 43 (12.5) |  | 100 (20.2) |  |  |
| 3+ y | 301 (87.5) |  | 394 (79.8) |  |  |

Abbreviations: SD, standard deviation; Δ, difference urban relative rural; s.e., standard error ^1^ Independent samples T-test. ^2^Land ownership was log transformed for these tests. Unlogged values are reported in the Table. ^3^Chi-square test. *p≤0.05, **p≤0.01, ***p≤0.001. ns, not significant

| \| Supplementary Table 4. Child anthropometry, stratified by sex \| \| \| \| \| \| \| \| \| --- \| --- \| --- \| --- \| --- \| --- \| --- \| --- \| \|  \| All children  **(n≥800)** \|  \| Female  **(n≥400)** \|  \| Male  **(n≥415)** \|  \| Difference^1^ \| \|  \| Mean (SD) \|  \| Mean (SD) \|  \| Mean (SD) \|  \| Δ (s.e.) \| \| Size at birth \|  \|  \| 2.7 (0.4)  48.5 (2.5)  33.4 (1.8)  -2.3 (1.0)  -0.2 (0.9)  -1.8 (1.0)  -0.2 (0.9)  -0.3 (1.0) \|  \| 2.8 (0.4)  49.1 (2.9)  34.0 (2.6)  -2.2 (1.1)  0.2 (1.0)  -1.6 (1.0)  0.2 (1.0)  -0.3 (1.1) \|  \| **-0.1 (0.03)****  **-0.5 (0.2)****  **-0.6 (0.1)*****  -0.1 (0.1)  **-0.4 (0.1)*****  -0.1 (0.1)  **-0.4 (0.1)*****  -0.04 (0.1) \| \| Weight (kg) \| 2.8 (0.4) \|  \|  \|  \| \| Length (cm) \| 48.8 (2.7) \|  \|  \|  \| \| Head circumference (cm) \| 33.7 (2.2) \|  \|  \|  \| \|  \|  \|  \|  \|  \| \| Size at 2 y \|  \|  \|  \|  \| \| Weight z-score  Conditional weight z-score \| -2.3 (1.1)  0.002 (1.0) \|  \|  \|  \| \| Height z-score \| -1.7 (1.0) \|  \|  \|  \| \| Conditional height z-score \| -0.001 (1.0) \|  \|  \|  \| \| BMI z-score \| -0.3 (1.1) \|  \|  \|  \| \| Head circumference (cm) \| 46.5 (1.4) \|  \| 45.8 (1.4) \|  \| 47.1 (1.3) \|  \| **-1.2 (0.1)***** \| \|  \|  \|  \|  \|  \|  \|  \|  \| \| Body composition at 8.5 y \|  \|  \|  \|  \|  \|  \|  \| \| Metric age (y) \| 8.4 (0.4) \|  \| 8.5 (0.4) \|  \| 8.4 (0.4) \|  \| 0.02 (0.03) \| \| Weight z-score \| -2.0 (1.0) \|  \| -2.1 (1.0) \|  \| -2.0 (1.1) \|  \| -0.05 (0.1) \| \| Height z-score \| -1.5 (0.9) \|  \| -1.5 (0.9) \|  \| -1.4 (0.9) \|  \| -0.1 (0.1) \| \| BMI z-score \| -1.6 (1.0) \|  \| -1.7 (0.9) \|  \| -1.6 (1.0) \|  \| -0.01 (0.1) \| \| Lean mass (kg)^2^ \| 17.3 (2.4) \|  \| 17.0 (2.3) \|  \| 17.6 (2.5) \|  \| **-0.7 (0.2)***** \| \| Fat mass (kg)^2^ \| 3.0 (1.6) \|  \| 2.9 (1.4) \|  \| 3.0 (1.7) \|  \| -0.1 (0.1) \| \| Head circumference (cm) \| 49.3 (1.5) \|  \| 48.7 (1.4) \|  \| 49.9 (1.3) \|  \| **-1.2 (0.1)***** \| \|  \|  \|  \|  \|  \|  \|  \|  \| \|  \| Frequency \|  \| Frequency (%) \|  \| Frequency (%) \|  \|  \| \| Sex \| 838 \|  \| 405 (48.3) \|  \| 433 (51.7) \|  \|  \| \|  \|  \|  \|  \|  \|  \|  \|  \|   Abbreviations: SD, standard deviation; Δ, difference female relative to male; s.e., standard error. ^1^Independent samples t-test. ^2^Sample size for lean and fat mass, all children n=626; female n=304; male n=322). **p≤0.01, ***p≤0.001. |
| --- | --- | --- | --- | --- | --- | --- | --- | --- | --- | --- | --- | --- | --- | --- | --- | --- | --- | --- | --- | --- | --- | --- | --- | --- | --- | --- | --- | --- | --- | --- | --- | --- | --- | --- | --- | --- | --- | --- | --- | --- | --- | --- | --- | --- | --- | --- | --- | --- | --- | --- | --- | --- | --- | --- | --- | --- | --- | --- | --- | --- | --- | --- | --- | --- | --- | --- | --- | --- | --- | --- | --- | --- | --- | --- | --- | --- | --- | --- | --- | --- | --- | --- | --- | --- | --- | --- | --- | --- | --- | --- | --- | --- | --- | --- | --- | --- | --- | --- | --- | --- | --- | --- | --- | --- | --- | --- | --- | --- | --- | --- | --- | --- | --- | --- | --- | --- | --- | --- | --- | --- | --- | --- | --- | --- | --- | --- | --- | --- | --- | --- | --- | --- | --- | --- | --- | --- | --- | --- | --- | --- | --- | --- | --- | --- | --- | --- | --- | --- | --- | --- | --- | --- | --- | --- | --- | --- | --- | --- | --- | --- | --- | --- | --- | --- | --- | --- | --- | --- | --- | --- | --- | --- | --- | --- | --- | --- | --- | --- | --- | --- | --- | --- | --- | --- | --- | --- | --- | --- | --- |
